# Marking the counterfactual: ERP evidence for pragmatic processing of German subjunctives

**DOI:** 10.3389/fnhum.2014.00548

**Published:** 2014-07-25

**Authors:** Eugenia Kulakova, Dominik Freunberger, Dietmar Roehm

**Affiliations:** Centre for Cognitive Research, University of SalzburgSalzburg, Austria

**Keywords:** conditionals, counterfactuals, EEG/ERP, linguistic mood, pragmatics, subjunctive

## Abstract

Counterfactual conditionals are frequently used in language to express potentially valid reasoning from factually false suppositions. Counterfactuals provide two pieces of information: their literal meaning expresses a suppositional dependency between an antecedent (*If the dice had been rigged…*) and a consequent (… *then the game would have been unfair*). Their second, backgrounded meaning refers to the opposite state of affairs and suggests that, in fact, the dice were not rigged and the game was fair. Counterfactual antecedents are particularly intriguing because they set up a counterfactual world which is known to be false, but which is nevertheless kept to when evaluating the conditional's consequent. In the last years several event-related potential (ERP) studies have targeted the processing of counterfactual consequents, yet counterfactual antecedents have remained unstudied. We present an EEG/ERP investigation which employed German conditionals to compare subjunctive mood (which marks counterfactuality) with indicative mood at the critical point of mood disambiguation via auxiliary introduction in the conditional's antecedent. Conditional sentences were presented visually one word at a time. Participants completed an acceptability judgment and probe detection task which was not related to the critical manipulation of linguistic mood. ERPs at the point of mood disambiguation in the antecedent were compared between indicative and subjunctive. Our main finding is a transient negative deflection in frontal regions for subjunctive compared to indicative mood in a time-window of 450–600 ms. We discuss this novel finding in respect to working memory requirements for rule application and increased referential processing demands for the representation of counterfactuals' dual meaning. Our result suggests that the counterfactually implied dual meaning is processed without any delay at the earliest point where counterfactuality is marked by subjunctive mood.

## Introduction

Counterfactual conditionals are frequently used in everyday language to convey potential dependencies between antecedents and consequents which have never been realized. A counterfactual conditional “*If Horst had not bought a pony, he would be miserable*” expresses a positive relation between Horst (suppositionally) not buying a pony and his (suppositional) misery. However, most people are able to infer from this sentence that Horst did in fact buy a pony (and is not miserable at all), even in absence of prior information about Horst (Thompson and Byrne, 2002). This notion of a dual meaning (suppositional and factual) has made the topic of counterfactual conditionals interesting for philosophers, linguists and psychologists alike and accounts for increasing attention of neurocognitive and psycholinguistic research on counterfactual sentence processing (Ferguson et al., 2008; Van Hoeck et al., 2010, 2012, 2013; Nieuwland and Martin, 2012; Nieuwland, 2012a,b; Urrutia et al., 2012; Kulakova et al., 2013).

In this recent line of neuroscientifically informed language research, counterfactual conditionals have been characterized by past tense and subjunctive mood. It has been argued before that it is primarily the pragmatics of subjunctive mood that marks the antecedent (i.e., the *if*-part) of the counterfactual conditional as factually false (Stalnaker, 1975). The subjunctive mood signals an inconsistency between the antecedent and the interlocutors' factual world knowledge. In contrast, the use of indicative mood (“*If Helga got a cat, then she is happy now.”*) in conditionals suggests compatibility between world knowledge and the proposition expressed by the antecedent. Helga might have bought a cat, or not, we don't know. Thus, even if the hearer does not already know the conflicting facts targeted by the counterfactual antecedent, she can infer them from the speaker's use of subjunctive mood. The factual information conveyed by counterfactuals (i.e., counterfactual antecedent falsity) has initially been identified as a presupposition phenomenon (Levinson, 1983; Fauconnier, 1994), although alternative accounts in terms of presuppositional and scalar implicatures have been presented recently (Verstraete, 2008; Leahy, 2011). All accounts agree that a pragmatic inference process is initiated to arrive at the (backgrounded) factual meaning of a counterfactual antecedent.

In a cognitive semantic form, the dual representation of counterfactuals was described within the mental spaces framework of Fauconnier (1994). Fauconnier introduces the concept of mental spaces, domains which are used to represent our mental (or subjectively accessible) representation of linguistic content. Counterfactual conditionals are considered as space builders, introducing new spaces as they go. Crucially, the parent space (usually representing the real world) and the counterfactually triggered mental space must contain incompatible information. The mental spaces account of counterfactuals captures the intuitive subjective experience during counterfactual sentence processing. However, it is difficult to approach the implications of this framework in an experimental manner. For example, the theory does not indicate whether a counterfactual is more difficult or costly to process than a sentence which does not open a new mental space. Furthermore, it does not indicate at which point of incremental sentence processing the counterfactual mental space is built up and for how long it is up-held.

Studies of online sentence processing have shown that subjects rapidly represent the factual state of affairs implied by counterfactual conditionals. Subjunctive but not indicative conditionals (*If A then B*) were shown to prime the implied factual event (¬A and ¬B), resulting in reading time benefits for its subsequent presentation (Santamaría et al., 2005). An eye-tracking study presented either counterfactual conditionals (*If the racing driver had won the race, …*) or factual sentences (*Because the racing driver had won the race, …*) as a context manipulation followed by either consistent or inconsistent declarative sentences (Ferguson, 2012). Importantly, the counterfactual continuation (*In the evening the racings driver's team* *celebrated*_inconsistent_/commiserated_consistent_) was either consistent or inconsistent with the implied factual information of the preceding counterfactual. The fact that subjects detected the inconsistency in the counterfactual condition (as indexed by more first-pass regressions out and back into the critical regions and longer total reading times of the pre-critical region) shows that subjects had access to the implied factual meaning of the counterfactual.

During the recent years an increasing amount of Event-Related Potential (ERP) studies has targeted counterfactual sentence processing. The neurocognitive approach to counterfactuals has primarily focused on the semantic N400 effect of world-knowledge violation in factual compared to counterfactual contexts (Ferguson et al., 2008; Nieuwland and Martin, 2012; Nieuwland, 2012a,b). Nieuwland and Martin (2012) demonstrated that the processing effort of a counterfactual consequent (as indexed by the N400 amplitude elicited by a critical word) is modulated by its consistency with the conditionals' antecedent rather than general world knowledge. This finding was furthermore demonstrated to hold for absolutely absurd consequences (*If dogs had gills, Dobermans would breathe under water*). The ERP elicited at the critical word was comparable to a factually true sentence (*Because fishes have gills, tuna breathe under water*). However, a pronounced N400 effect emerged at the critical word when the licensing counterfactual antecedent was omitted (Nieuwland, 2012b). This suggests that the (factually false) propositions expressed by counterfactual antecedents are quickly incorporated into the discourse, licensing subsequent conclusions which are factually false but consistent with a locally established context.

These results taken together suggest that both the factual and suppositional meaning of a counterfactual are accessed rapidly, as expected from a double representation account. If this is true, it should be possible to observe effects of increased processing costs arising from the dual counterfactual representation at the point of linguistic mood disambiguation, since counterfactuality is for the first time marked by subjunctive mood use in the conditionals' antecedent. However, to our knowledge no study has yet investigated the immediate effect of counterfactuality introduction in the antecedent of a counterfactual conditional. The reason why research has neglected counterfactual antecedents processing for a long time may lie in the fact that until now ERP studies of counterfactuals have relied on factual “Because … ” constructions as the contrast conditions for counterfactuals, which makes it difficult to interpret any effect at the auxiliary because up to the critical point sentences already differ in length and wording. A recent proposal from imaging research on counterfactual sentence processing suggests that the hypothetical conditional as marked by indicative mood provides a more appropriate control condition, paralleling the counterfactual in the suppositional ontological status as well as the If-then structure (Kulakova et al., 2013). The only remaining difference between these conditions is linguistic mood, introducing the counterfactuals' antagonism to factual events (see also Stewart et al., 2009).

In the present study we investigated the neural consequences of counterfactuality introduction in the antecedent of German conditionals. We compared the ERPs elicited by auxiliary verbs (i.e., grammatical entities that carry information about linguistic mood) marking subjunctive and indicative mood. To our knowledge this was the first attempt to experimentally pinpoint the process of counterfactuality marking in a subjunctive antecedent. We used an electroencephalographic (EEG) measure which is very sensitive to fast, automatic, and unconscious processes during sentence processing and provides detailed information about the time-course of neuronal events. We expected to observe an ERP difference reflecting the process of counterfactual implication detection leading to a dual representation in subjunctive compared to indicative mood.

Opposite to the English language, German allows the implementation of this comparison in a very straightforward way. Subjunctive mood in past tense has a distinct morphology that, contrary to English, does not comprise a double layer of past tense which inevitably results in sentence length differences. Another advantage of German syntax that facilitates the investigation of mood-disambiguation at this early point comes from the fact that the mood-disambiguating auxiliary takes the final position in the antecedent where propositional content is already conveyed. See an example of our stimulus material in Table 1. Both conditions are identical in the first four words which set up the suppositional proposition of rigged dice (Wenn die Würfel gezinkt …). The next word is the auxiliary verb which introduces linguistic mood: In the counterfactual condition “wären” marks subjunctive mood and allows the reader to infer that although it is supposed that the dice were rigged (suppositional meaning), they in fact were not (factual meaning). In the indicative condition “waren” introduces the suppositional reading only, allowing no inferences about the factual state of affairs.

**Table 1:** **Example of German stimulus sentences (critical word underlined) with literal and analogous English translations**.

**Subjunctive (counterfactual)**
Wenn die Würfel gezinkt wären, dann wäre das Spiel fair/unfair.
*If the dice rigged were*_subjunctive_, *then would be the game fair/unfair*.
*“If the dice had been rigged, then the game would have been fair/unfair.”*
**Indicative**
Wenn die Würfel gezinkt waren, dann war das Spiel fair/unfair.
*If the dice rigged were*_indicative_, *then was the game fair/unfair*.
*“If the dice were rigged, then the game was fair/unfair.”*

We incorporated our experimental manipulation of linguistic mood in an acceptability judgment and probe detection task, so that consciously inferring the counterfactually implied factual information was not required for correct task performance. This was intended in order to investigate the automatic (i.e., task-irrelevant) effect of counterfactual sentence processing occurring at the point of mood disambiguation via the introduction of the auxiliary in the conditionals' antecedent. We used German conditional sentences describing causal and definitional regularities which have been demonstrated to trigger a counterfactual reading when presented in subjunctive mood (Thompson and Byrne, 2002). We measured ERPs elicited at the critical antecedent-final auxiliary but also report the effects of the sentence-final manipulation of truth. Since the targeted process of counterfactuality processing was semantic/pragmatic in nature we did not expect to observe the critical effect to occur early, but rather in time-window where effects of semantic manipulations (i.e., N400, LAN, P600) are usually reported. Potential effects of visual dissimilarity of the compared auxiliaries should, if occurring at all, manifest in rather early differences starting at around 150 ms post onset (Sauseng et al., 2004).

## Materials and methods

### Participants

Sixteen German native speakers (7 female; mean age 23.5; age range 18–36 years) participated in the ERP study. All subjects were right-handed according to an adopted German version of the Edinburgh Inventory (Oldfield, 1971) and had normal or corrected-to-normal vision. Subjects gave informed consent before the experiment and were paid 20€ for participation. All methods conformed to the Code of Ethics of the World Medical Association (Declaration of Helsinki) (see Mayrhauser et al., 2014).

### Materials

Stimulus material consisted of 52 sets of German conditionals describing social, physical or biological regularities. The experimental factor of interest was Mood (two levels: indicative, subjunctive). Furthermore, the sentence-final content word varied as a function of Truth (two levels: true, false) in order to balance acceptability. Two auxiliary verbs forms, *haben* (to have) and *sein* (to be), were used in equal amounts to make the stimuli less monotonous. Their past tense plural forms are visually maximally similar between past indicative and subjunctive (*hatten*/*hätten, waren*/*wären*), only differing in the diacritic umlaut on the first vowel. Since this could have added an effect of auxiliary due to possible differences in naturalness, cloze or word frequency effects, we carried out additional tests to specify these parameters and also included auxiliary type (factor Aux, two levels: to be, to have) in further analyses. Each of the 52 thematic vignettes was presented twice per subject in different counterbalanced conditions, resulting in 104 items in total. Additionally, the experiment contained 208 declarative (non-conditional) filler sentences which were also balanced in acceptability (e.g., *No vegetarian likes eating roast/vegetables)*.

#### Naturalness rating

To investigate possible effects of perceived naturalness, we conducted a rating-task of our stimulus material in respect to the four auxiliaries employed. In an internet-based survey 50 participants (none of which took part in the EEG-experiment) rated the auxiliaries of the antecedents (consequents were not presented to avoid confounding effects of plausibility) for their naturalness on a 7 point scale. A 2 × 2 repeated measures ANOVA with the factors Mood and Aux revealed a main effect of Mood [*F*_(1, 48)_ = 163.2, *p* < 0.001] and a Mood × Aux interaction [*F*_(1, 48)_ = 14.7, *p* < 0.001]. Resolving the interaction by Aux indicated that naturalness ratings were higher for subjunctive both for “to be” [*T*_(48)_ = 4.6, *p* < 0.001] and “to have” [*T*_(48)_ = 12.8, *p* < 0.001]. See Table 2 for detailed behavioral results.

**Table 2:** **Stimulus properties and behavioral results split by mood (indicative/subjunctive) and auxiliary (to be/to have)**.

			**Indicative**		**Subjunctive**	
			**To be**	**To have**	**To be**	**To have**
Frequency			711	363	166	242
Naturalness			4.1 (1.7)	3.5 (1.2)	5.3 (1.4)	5.7 (0.8)
Cloze			0	0	0.08	0.18
Acceptability judgment	True	% accepted	95.1 (7)	95.1 (7)	94.7 (8)	93.1 (7)
		RT in ms	577 (245)	712 (260)	620 (286)	712 (245)
	False	% accepted	4.4 (7)	3.5 (7)	5.7 (8)	4.2 (11)
		RT in ms	606 (289)	742 (308)	730 (326)	724 (341)
Probe detection	True	% detected	96.9 (5)	99.6 (2)	98.7 (3)	99.0 (4)
		RT in ms	884 (243)	853 (254)	852 (233)	946 (290)
	False	% detected	99.5 (2)	99.1 (2)	98.4 (3)	99.5 (2)
		RT in ms	922 (314)	994 (234)	1026 (300)	945 (248)

*Frequencies per million, offline ratings of naturalness (N = 50) and cloze probability (N = 58). Online acceptability and probe detection responses (N = 16) with corresponding reaction times (RT) for true and false sentences. Standard deviations are given in parentheses*.

#### Completion task

We also tested predictability differences between indicative and subjunctive auxiliaries employed in our task. In an internet-based survey a cohort of 58 subjects (none of which took part in the EEG-experiment or carried out the naturalness rating) completed 30 randomly selected sentences from our stimulus material truncated before the critical auxiliary. The subjunctive past tense continuation was chosen with a mean probability of 0.13, the indicative past auxiliary was almost never chosen (<0.01). Instead, subjects chose the more colloquial future or present indicative forms of the respective auxiliary, but no other word forms. See Table 2 for detailed cloze values. Table 2 also presents the frequencies (per million) of the employed auxiliaries derived from the SUBTLEX-DE database (Brysbaert et al., 2011).

### Procedure

Trials began with a fixation cross presented for 1500 ms. After 900 ms blank screen, sentences were presented word-by-word with a single word duration of 400 ms (nominal phrases 500 ms) and an inter-stimulus interval of 200 ms. The critical words were presented with a comma, the last word of each sentence was presented with a period. Each sentence was followed by an acceptability judgment where a green question mark prompted subjects to give a yes/no acceptability judgment. This was followed by a probe detection task in which subjects judged if a single displayed word had been presented in the previous sentence. Maximal response duration for each task was 2000 ms. Participants responded by pressing the left or right shift-key on a computer keyboard. The assignment of response buttons was counterbalanced across participants. Inter-trial interval was 800 ms. Experimental and filler sentences (312 trials in total) were pseudo-randomized and presented in 6 blocks with 52 different items per block. Half of the participants read the sentences in reverse order to control for sequence effects.

### EEG recording and preprocessing

EEG was recorded from 25 Ag/AgCl electrodes mounted in an elastic cap (Easy Cap International, Herrsching-Breitbrunn, Germany) according to the 10/20 system (Jasper, 1958). Electrodes included: AFz, Fz, FCz, Cz, CPz, Pz, POz, F7/8, F3/4, FC5/6, FC1/2, CP5/6, CP1/2, P7/8, P3/4, O1/2. The EEG-signal was sampled at 500 Hz with a low-pass filter at 250 Hz and a software notch filter (50 Hz). Data were recorded with respect to the left mastoid reference and electrode AFz served as ground electrode. The horizontal electro-oculogramm (HEOG) was recorded from electrodes at the outer canthus of each eye. The vertical electro-oculogramm (VEOG) was recorded from electrodes placed above and beneath the left eye. Scalp impedances were kept below 5 kΩ.

Offline, all electrodes were re-referenced to the average activity of the left and right mastoids, before an independent component analysis (ICA) was applied to correct ocular artifacts. After correction, remaining artifacts (EOG, movement, technical) were marked manually. In total, 5.1% of indicative 7.7% of subjunctive trials had to be rejected. The signal was band-pass filtered from 0.3–20 Hz in order to exclude slow drifts and then was segmented into epochs from −300 to 800 ms around the critical words. The signal was averaged for each condition and each participant before grand averages were computed across all participants.

For the statistical analysis of acceptability and probe detection, mean acceptability (% accepted) and probe detection accuracy (% detected) and the corresponding reaction times were subjected to repeated measure ANOVAs with the factors Mood (indicative/subjunctive), Truth (true/false) and Aux (to have/to be).

**Figure 1:**
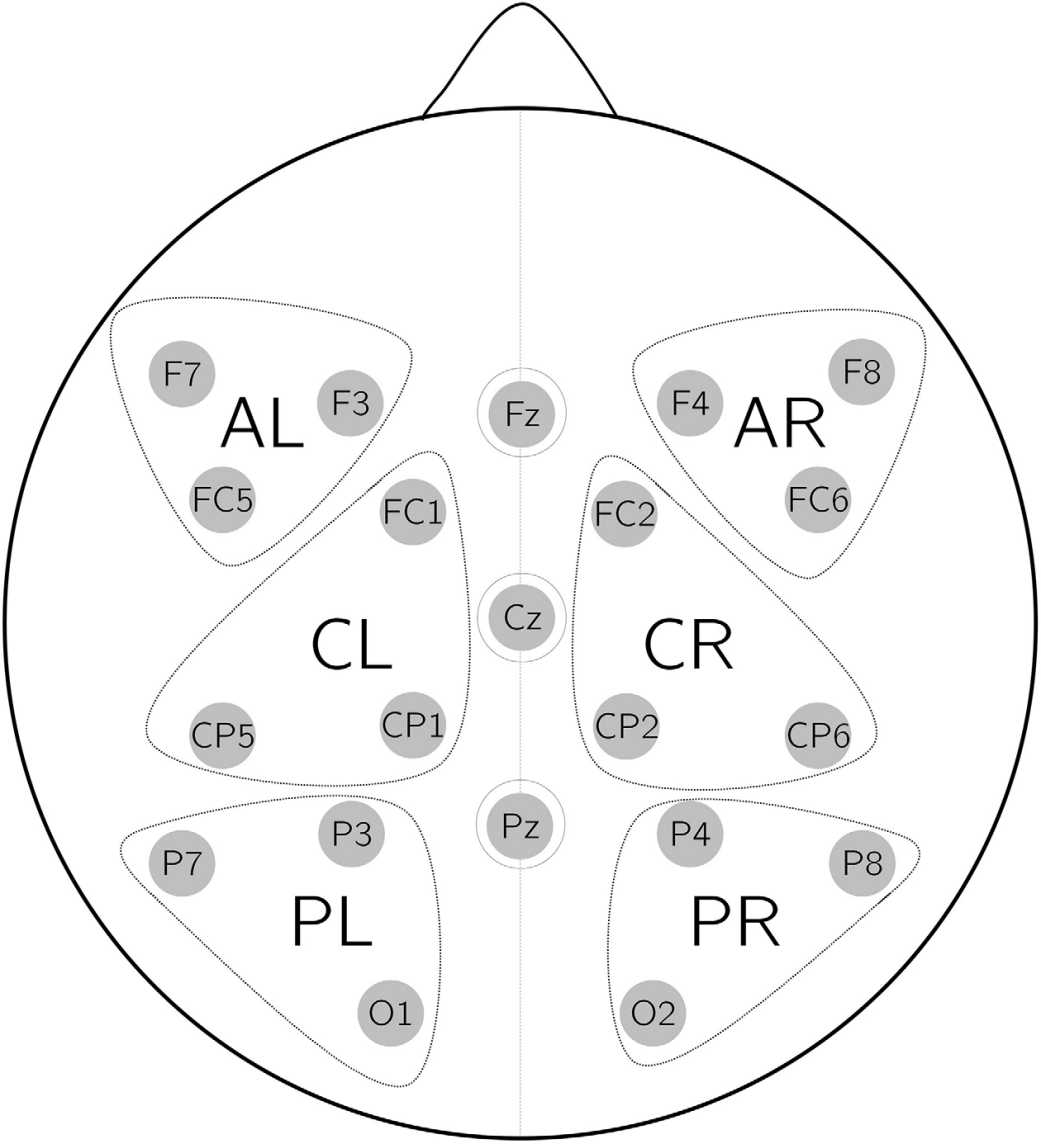
**Topography of the six regions of interest (AL, left anterior; AR, right anterior; CL, left central; CR, right central; PL, left posterior; PR, right posterior)**.

Based on visual inspection statistical analyses of the ERPs were carried out in two time windows: 200–400 ms and 450–600 ms. For both time windows the mean amplitude was computed per subject, condition, and electrode. Mean amplitude values were submitted to a 2 × 2 × 6 repeated measures ANOVA with the factors Mood, Aux and Roi (regions of interest). The factor Truth was omitted because it played no role at the critical position. Six ROIs were calculated from the mean value of three electrodes each: anterior left (AL; F3, F7, FC5), anterior right (AR; F4, F8, FC6), central left (CL; FC1, CP1, CP5), central right (CR; FC2, CP2, CP6), posterior left (PL; P3, P7, O1), and posterior right (PR; P4, P8, O2), see Figure 1 for the topography of the ROIs. Another 2 × 2 × 3 ANOVA was performed with the factor Mid (midline electrodes) where three midline electrodes were treated as separate ROIs (Fz, Cz, Pz). *P*-values with more than one degree of freedom in the numerator were corrected according to Greenhouse-Geisser. Only significant interactions were resolved. To disentangle the effects emerging in the statistics pairwise comparisons were computed and are reported at an FDR-corrected threshold (Benjamini and Hochberg, 1995). Effects of Aux, Roi, and Mid are presented but not discussed unless there is an interaction with Mood.

Given the employed target manipulation (Steinhauer and Drury, 2012) of the critical auxiliary, no systematic differences between the baseline intervals were expected. Instead of using a baseline correction which could carry over transient signal differences from the baseline interval to the critical time window, we employed a filter which serves to remove unsystematic pre-stimulus differences due to slow drifts (below 0.3 Hz). The baseline interval was checked for significant differences in steps of 50 ms from −200 to 100 ms around the onset of the critical word to demonstrate the absence of baseline differences.

As a further check of data quality the sentence-final words were analyzed in the time windows of 300–500 ms and 500–800 ms with the expectation to observe an N400/P600 effect commonly known to reflect semantic violations. All additional analyses are reported in the Supplementary Material.

## Results

### Behavioral results

#### Acceptability judgment

Subjects rated true sentences as acceptable and false sentences as inacceptable. The 2 (Mood) × 2 (Truth) × 2 (Aux) repeated measures ANOVA revealed only a main effect of Truth [*F*_(1, 15)_ = 2005.6, *p* < 0.001] with higher acceptability rates for true compared to false sentences. The comparison of reaction times for acceptability ratings showed a main effect of Aux [*F*_(1, 15)_ = 26.3, *p* < 0.001] and a significant Aux × Mood interaction [*F*_(1, 15)_ = 4.8, *p* < 0.05]. Resolving the interaction by Aux indicated that only in the auxiliaries of “to be” subjunctive mood led to significantly increased reaction times [*T*_(15)_ = 2.5, *p* < 0.05]. A possible reason for this effect is a larger length difference between indicative and subjunctive sentence-final words after “to be” (difference of 0.24 characters) than after “to have” (difference of 0.05 characters).

#### Probe detection

Performance in the probe detection task was almost perfect. A 2 × 2 × 2 repeated measures ANOVA showed neither main effects nor interactions of the explanatory variables. The analysis of probe detection speed showed a main effect of Truth [*F*_(1, 15)_ = 12.2, *p* < 0.01] and an Aux × Mood × Truth interaction [*F*_(1, 15)_ = 6.7, *p* < 0.05]. Resolving the interaction by Aux revealed a significant Mood × Truth interaction for “to have” [*F*_(1, 15)_ = 8.8, *p* < 0.01] but not for “to be” [*F*_(1, 15)_ = 2.8, *p* = 0.11]. Further paired comparisons showed that reaction times in true “to have” sentences were faster only in indicative [*T*_(15)_ = 3.9, *p* < 0.01] but not subjunctive mood [*T*_(15)_ < 1]. Detailed behavioral responses with corresponding reaction times can be found in Table 2.

### ERP results

Detailed test-statistics are shown in Table 3. The 2 (Mood) × 2 (Aux) × 6 (Roi) repeated measure ANOVA for the lateral electrodes in the 200–400 ms time window revealed that subjunctive mood elicited a significantly more positive deflection compared to indicative as indicated by a main effect of Mood. Midline analyses in the same time window showed significant main effects of Mood and Mid and a significant Aux × Mid interaction. However, resolving this interaction did not reveal any electrodes at which the Aux effect reached significance [*Fz*: *T*_(15)_ = 1.34, *p*_FDR_ = 0.60; *Cz*/*Pz*: *T*_(15)_ < 1].

**Table 3:** **Test-statistics of the ERPs elicited by the critical auxiliaries in two time windows**.

	**Time window**							
	**200–400 ms**				**450–600 ms**			
**ANOVA lateral electrodes**	***df***	***F***	***p***	***sig***	***df***	***F***	***p***	***sig***
Mood	1, 15	26.34	<0.001	***	1, 15	4.36	<0.05	*
Aux	1, 15	<1	0.97		1, 15	<1	0.96	
Roi	5, 75	15.27	<0.001	***	5, 75	5.58	<0.01	**
Aux × Mood	1, 15	<1	0.46		1, 15	1.33	0.27	
Mood × Roi	5, 75	2.46	0.08		5, 75	3.07	<0.05	*
Aux × Roi	5, 75	2.53	0.09		5, 75	3.06	<0.05	*
Aux × Mood × Roi	5, 75	<1	0.55		5, 75	1.70	0.18	
**Resolution by ROI**					***df***	***T***	***p_FDR_***	***sig_FDR_***
Mood (AL)					15	3.07	<0.05	*
Mood (AR)					15	1.89	0.12	
Mood (CL)					15	2.23	0.12	
Mood (CR)					15	1.86	0.12	
Mood (PL)					15	1.36	0.23	
Mood (PR)					15	<1	0.59	
**ANOVA midline electrodes**	***df***	***F***	***p***	***sig***	***df***	***F***	***p***	***sig***
Mood	1, 15	15.42	<0.001	***	1, 15	4.70	<0.05	*
Aux	1, 15	<1	0.57		1, 15	<1	0.78	
Mid	2, 30	19.18	<0.001	***	2, 30	13.71	<0.001	***
Aux × Mood	1, 15	<1	0.97		1, 15	1.34	0.27	
Mood × Mid	2, 30	1.81	0.19		2, 30	3.90	<0.05	*
Aux × Mid	2, 30	3.89	<0.05	*	2, 30	4.24	<0.05	*
Aux × Mood × Mid	2, 30	<1	0.55		2, 30	<1	0.66	
**Resolution by MID**					***df***	***T***	***p_FDR_***	***sig_FDR_***
Mood (Fz)					15	3.58	<0.01	**
Mood (Cz)					15	1.99	0.10	
Mood (Pz)					15	<1	0.36	

*Main effects and interactions of the performed ANOVAs and resolution of significant interactions with Mood by region of interest (ROI) and midline (MID) electrodes, respectively. AUX, auxiliary; AL, anterior left; AR, anterior right; CL, central left; CR, central right; PL, posterior left; PR, posterior right*.

**Figure 2:**
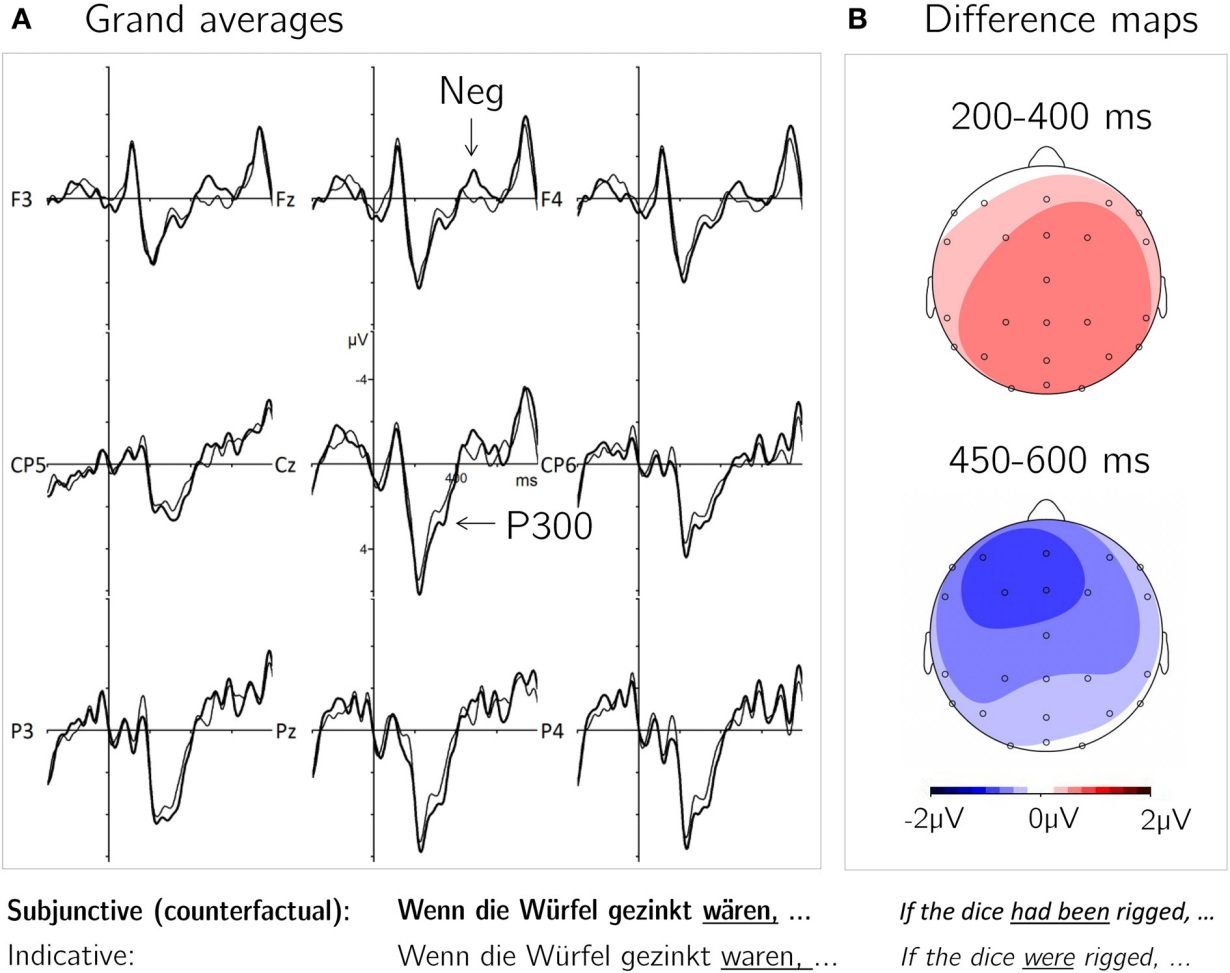
**ERP Results**. **(A)** Grand average waveforms time-locked to the antecedent-final auxiliary disambiguating mood: subjunctive (thick line) and indicative (thin line) mood. **(B)** Difference maps for the contrast subjunctive > indicative in the relevant time windows illustrate the topography of the effects.

In the 450–600 ms time window the analysis of lateral ROIs showed main effects of Mood and Roi and significant Mood × Roi and Aux × Roi interactions. Former indicated that the stronger negativity elicited by subjunctive mood was most pronounced at left anterior and central electrodes and absent in right and posterior regions. Resolving the Aux × Roi interaction did not reveal any ROI which showed a significant effect auf Aux [AL/CL/CR/PR: *T*_(15)_ < 1; AR: *T*_(15)_ = 1.31, *p*_FDR_ = 0.67; PL: *T*_(15)_ = 1.27, *p*_FDR_ = 0.67]. The midline ANOVA showed a main effect of Mood and Mid with a significant Mood × Mid and Aux × Mid interaction. The former indicated a frontal distribution of the mood effect (i.e., stronger negativity for subjunctive compared to indicative). Resolving the latter did not disclose any electrodes at which the effect of Aux reached significance [Fz: *T*_(15)_ = 1.12, *p*_FDR_ = 0.82; Cz/Pz: *T*_(15)_ < 1]. Figure 2 shows the grand average ERP waveforms at selected electrode sites for both levels of the experimental condition Mood, time locked to the critical point of mood disambiguation via auxiliary introduction.

The analysis of the sentence-final content word showed a clear N400/P600 pattern which was expected given our manipulation of truth and the acceptability rating task. None of the pre-critical time-windows showed effects of Mood or Aux. Detailed test-statistics of the additional analyses can be found in the Supplementary Material.

## Discussion

The present ERP experiment examined conditional sentence processing at the critical point of linguistic mood introduction in the antecedent of conditionals. We expected to observe an ERP effect reflecting the process of counterfactuality marking in subjunctive compared to indicative antecedents. This implication detection process is required to establish a dual representation of counterfactual meaning and was expected to be automatic and thus to occur in a task which did not involve explicit factual implication computation. In accordance with our expectations we found a positive deflection for subjunctive compared to indicative auxiliaries in the time-window between 200–400 ms, followed by a left frontal negativity in a time window of 450–600 ms.

### Prediction-related P300

We interpret the early centro-parietal positivity for subjunctive relative to indicative auxiliaries as an instance of the prediction-related P300, since it has a similar time-course and distribution as described in previous studies (Roehm et al., 2007; Molinaro and Carreiras, 2010; Vespignani et al., 2010). This ERP component has been shown to reflect the integration of an expected lexical element into the prior context. When there is a successful match of the predicted and the actual input, a P300 is generated. Cloze values and naturalness-ratings support the conjecture: in our stimulus set the subjunctive continuation was anticipated with a greater likelihood and rated to sound more natural than the indicative auxiliary. Thus, the antecedent's context triggered a significantly stronger expectation of the subjunctive auxiliaries, the integration of which elicited a P300 effect in comparison to the less expected indicative auxiliary. This effect arises from the cloze values of the employed stimuli but should not be replicated if cloze values between indicative and subjunctive auxiliaries were matched.

### Left anterior negativity and definiteness

Interestingly, the negative frontal effect for subjunctive vs. indicative auxiliaries shows that, although locally predicted, counterfactuals pose increased processing demands. Since lexical prediction presumably does not go beyond semantics, the pragmatic implication carried by the subjunctive word forms still required additional processing. The observed transient negativity from 450–600 ms for subjunctive auxiliaries bears topographical and temporal resemblance to the left anterior negativity (LAN) effect as often observed for function words as employed in our study. Generally, the LAN has been proposed to index increased syntactic working memory demands imposed by un-canonical sentence structure (Rösler et al., 1998; Matzke et al., 2002). Recently, a transient LAN effect was observed for regular compared to irregular German past participles and was thus proposed as a marker of syntactic rule application (Krott and Lebib, 2012). In our task, however, the employed correct form auxiliaries did not differ functionally between indicative and subjunctive mood (neither in terms of inflection, conveyed syntactic complexity nor task demands). Thus, the increased working-memory demand indexed by the observed negativity effect is most parsimoniously explained as stemming from the pragmatic process of counterfactual implication detection, mirroring the application of a pragmatic rather than syntactic rule.

A recent finding supports the link between the present results and the topic of implication detection in terms of presupposition processing. German definite articles in noun-phrases have been reported to trigger a significantly stronger negative deflection than indefinite articles in the time-window of 400–700 ms with a left anterior topography very similar to the one presently observed (Schumacher, 2009). This effect has been argued to reflect increased working memory demands for the integration of a definite referent into discourse. Notably, definite articles and counterfactuals have both been proposed to be presupposition triggers (Levinson, 1983), since both elicit a pragmatic inference process in order to make sense of the use of a specific expression given the shared beliefs of speaker and hearer. The presupposition attached to the use of the definite article “the” introduces the assumption that the referent of the noun following “the” exists and is contextually salient. This parallels the process by which subjunctive mood marks the proposition of the antecedent as false, simultaneously implying its opposite as factually true, thus referring to a specific factual event. Indefinite articles and indicative mood, on the other hand, do not convey any definiteness about the introduced proposition, and thus lack reference to any specific discourse entity. Taken together, these results suggest that the amplitude of the observed negativity is sensitive to the pragmatic process of counterfactuality marking via implication detection.

### Effects of frequency and cloze

Since the frequencies of the employed subjunctive auxiliaries are generally lower than those marking indicative mood, an effect of word frequency could be expected to drive our main finding. However, the LAN reported for closed-class words has been shown to be frequency independent (Brown et al., 1999; Molinaro et al., 2008). Only one study reported a more pronounced negativity for very highly frequent compared to high, medium and low frequency words (Münte et al., 2001). Thus, our results show if any, then the opposite pattern of a frequency effect. There is therefore no indication that the negativity effect reported in our data stems from the frequencies of the employed auxiliaries.

A similar line of argumentation applies to a possible confounding effect of cloze value and rated naturalness. Less expected words are usually associated with a stronger negativity compared to highly anticipated continuations of a sentence in terms of an N400 effect (Federmeier et al., 2007). Since in our stimuli the indicative auxiliary received lower cloze values and naturalness ratings our main finding of a stronger negativity for subjunctive auxiliaries is contrary to an expected effect driven by cloze. Thus, the confoundation with cloze/naturalness and frequency in our material rather leads to an underestimation of the observed effect associated with processing counterfactuals than being an alternative explanation for it. Since the N400 is known to be most pronounced over central and posterior electrode sites (e.g., Kutas and Federmeier, 2011) such an overlap would be strongest at these regions and thus could be shifting the observed negativity effect of counterfactuality toward anterior sites. In this case further studies with cloze-values matched for mood might find stronger negativity effects of counterfactuality with broader distributions than presently observed.

### Referentially induced frontal negativity

Another stance toward the dual meaning of counterfactuals is referential plurality. Two meanings, one literal suppositional and the other backgrounded factual, are conveyed at the point where subjunctive mood suggests a counterfactual reading of the antecedent's proposition. Indicative antecedents, on the other hand, carry only their literal suppositional meaning. This links the present findings to literature on referential ambiguity where processing pronouns with more than one definite referent in the salient discourse space has been shown to elicit sustained frontal negative shifts emerging at around 300 ms after stimulus onset (Van Berkum et al., 2007). There are three interpretations of this so called Nref-effect. The negativity could reflect merely noticing the ambiguity, the attempt to resolve it in terms of inference processes, or the attempt to simultaneously keep two competing referential interpretations in working memory, an interpretation which relates the Nref to the sustained LAN effect reflecting increased working memory demands. Since counterfactuals' referential plurality probably does not introduce a sustained ambiguity which has to be resolved at some later point, our finding of a transient negativity for subjunctive auxiliaries might be related to the Nref but rather represent the identification and computation of referential plurality of counterfactuals in terms of their dual meaning.

Taken together, we propose that the increased processing demand for subjunctive mood as indexed by the observed negativity effect reflects the process of counterfactuality marking in terms of implication detection which is relevant for a representation of the counterfactuals' dual meaning. This process appears on an implicit, task-independent level of sentence processing.

### N400/P600 effects of semantic acceptability

Although not in the focus of our investigation, we additionally analyzed the ERPs elicited by the sentence-final content word which established the manipulation of truth in our task. Behavioral results indicate that the acceptability ratings were given in accordance to our truth manipulation. Similarly, the ERPs results show a clear N400/P600 signature for content-words which rendered the sentence false in comparison to those which rendered it true. “False” words were semantically incongruent with preceding context and therefore no contextual facilitation of the lexical entry could be initialized. The N400 reflects the increased costs of the lexico-semantic acccess of incongruent compared to congruent sentence-final words (Kutas and Federmeier, 2011). Furthermore, it has been shown that acceptability rating tasks make it more likely for semantic violations to elicit a P600 effect following the N400 (Kuperberg, 2007), which is what we observe in our data. Our N400 results are consistent with previous findings of Nieuwland (2012b) and Nieuwland and Martin (2012) which indicate that the neurocognitive effect of establishing truth-value in counterfactuals depends on the consistency with the preceding antecedent rather than on general world knowledge. Consistently, we did not observe any modulation of the N400 effect by linguisitc mood as indicated by the absence of an interaction between these factors.

## Conclusion

Our study is to our knowledge the first investigation of counterfactual antecedents using ERPs. We observed that subjunctive auxiliaries which mark counterfactuality in antecedents elicited an early positivity which we interpret as a prediction-related P300 effect, mirroring the increased expectation of a subjunctive compared to an indicative continuation of the antecedents in our stimulus set. More importantly, we observed a negativity effect with a left frontal distribution for subjunctive compared to indicative auxiliaries in the time-window of 450–600 ms. We propose that this effect indicates increased processing demands of counterfactual antecedents triggered by the establishing of the counterfactuals' dual meaning. Our results demonstrate that the dual meaning arises without any delay at the earliest point where counterfactuality is marked.

This result demonstrates the existence of a neural correlate of subjunctive mood processing. This first evidence of a neural marker for counterfactuality will allow further studies to explore the sensitivity to counterfactuality in different contexts and age groups. The negativity effect may for example serve as a developmental precursor for counterfactual sentence comprehension capabilities and should precede children's ability to reason correctly from a counterfactual antecedent without committing the reality bias or resorting to basic conditional reasoning which is characterized by neglecting the factually implied specific circumstances of counterfactuals (Rafetseder and Perner, 2014). Similar applications are conceivable for patients with lesions or clinical populations.

An appealing speculation is that the observed negativity effect is associated with the initiation of inhibitory processes necessary to overcome the reality-bias in counterfactual reasoning. This would lead to the prediction that the strength of the effect depends on contextual salience of factually implied events because more salient stimuli need more inhibition. This motivates subsequent studies which employ absurd counterfactual antecedents as used by Ferguson and Sanford (2008) or Nieuwland (2012b) which explicitly violate factual knowledge (e.g., “*If dogs had gills…*”), with the expectation to observe even stronger integration costs. Furthermore, correlational investigations of negativity amplitude and duration with individual executive function scores appear promising.

An alternative speculation is that the negativity observed in our study is an index of a pragmatic violation in terms of a process comparable to presupposition failure. The greatest share of literature reporting a transient LAN (which is, however, followed by a late positivity which is not observed here) comes from ERP studies of morpho-syntactic violations (Kutas and Hillyard, 1983). Although our data do not show a significant late positivity effect and counterfactual antecedents are rated as sounding rather natural, it could be tentatively proposed that the use of subjunctive mood in our materials constitutes a pragmatic violation, since no contradictory factual knowledge is salient to license its use. In that case more salient factual events which are implicitly referred to by the counterfactual antecedent should rather decrease the amplitude of the negativity effect, since they operate as a licensor and facilitate reference assignment. More studies with manipulations of context and task that take into account individual difference in pragmatic sensitivity are required to resolve this interesting issue.

However, all conceivable explanations support an interpretation of the observed negativity effect for subjunctive antecedents in terms of a pragmatic effect related to inference processes associated with counterfactuality marking in order to arrive at the counterfactuals' dual meaning.

### Conflict of interest statement

The authors declare that the research was conducted in the absence of any commercial or financial relationships that could be construed as a potential conflict of interest.
