# Supplementary material for: Marking the counterfactual: ERP evidence for pragmatic processing of German subjunctives
Source: Front Hum Neurosci. 2014 Jul 25;8:548. doi: 10.3389/fnhum.2014.00548 (PMC4110946; doi:10.3389/fnhum.2014.00548)
Supplement: Supplementary file 1 [file DataSheet1.PDF]

Supplementary material

1. *Sentence-final content word*

True: Wenn die Würfel gezinkt waren/wären, dann war/wäre das Spiel unfair.
If the dice were/had been rigged, then the game was/would have been unfair.
False: Wenn die Würfel gezinkt waren/wären, dann war/wäre das Spiel fair.
If the dice were/had been rigged, then the game was/would have been fair.

	Time window							
	300-500 ms				600-800 ms			
	df	*F*	*p*	*sig*	*df*	*F*	*p*	*sig*
ANOVA lateral electrodes								
MOOD	1,15	< 1	.91		1,15	1.59	.22	
TRUTH	1,15	10.86	< .01	**	1,15	19.27	< .001	***
ROI	5,75	6.98	< .01	**	5,75	5.56	< .01	**
MOOD*TRUTH	1,15	< 1	.40		1,15	4.90	< .05	*
MOOD*ROI	5,75	2.52	.08		5,75	< 1	.61	
TRUTH*ROI	5,75	4.36	< .01	**	5,75	13.01	< .001	***
MOOD*TRUTH*ROI	5,75	1.02	.36		5,75	2.72	.08	
Resolution by ROI	*df*	*T*	p_{FDR}	sig_{FDR}	*df*	*T*	p_{FDR}	sig_{FDR}
TRUTH (AL)	15	< 1	.55		15	1.63	.14	
TRUTH (AR)	15	2.05	.07		15	1.75	.14	
TRUTH (CL)	15	2.82	< .05	*	15	4.12	< .01	**
TRUTH (CR)	15	3.73	< .01	**	15	4.73	< .01	**
TRUTH (PL)	15	4.40	< .01	**	15	4.92	< .01	**
TRUTH (PR)	15	3.98	< .01	**	15	6.06	< .01	**
ANOVA midline electrodes	*df*	*F*	*p*	*Sig*	*df*	*F*	*p*	*sig*
MOOD	1,15	< 1	.63		1,15	< 1	.40	
TRUTH	1,15	6.46	< .05	*	1,15	18.83	< .001	***
MID	2,30	4.10	< .05	*	2,30	4.17	< .05	*
MOOD*TRUTH	1,15	< 1	.56		1,15	7.97	< .01	**
MOOD*MID	2,30	8.07	< .01	**	2,30	< 1	.89	
TRUTH* MID	2,30	7.72	< .01	**	2,30	18.96	< .001	***
MOOD*TRUTH* MID	2,30	< 1	.36		2,30	1.65	.22	
Resolution by MID	*df*	*T*	p_{FDR}	sig_{FDR}	*df*	*T*	p_{FDR}	sig_{FDR}
TRUTH (Fz)	15	< 1	.55		15	1.85	.08	
TRUTH (Cz)	15	3.03	< .05	*	15	4.42	< .001	***
TRUTH (Pz)	15	2.92	< .05	*	15	5.68	< .001	***

Table 1. Test-statistics of the ERPs elicited by the sentence-final content words in two time windows corresponding to the N400 and P600 effects. Main effects and interactions of the performed ANOVAs and resolution of significant interactions with Truth by region of interest (ROI) and midline (MID) electrodes, respectively. AUX = auxiliary, AL = anterior left, AR = anterior right, CL = central left, CR = central right, PL = posterior left, PR = posterior right.

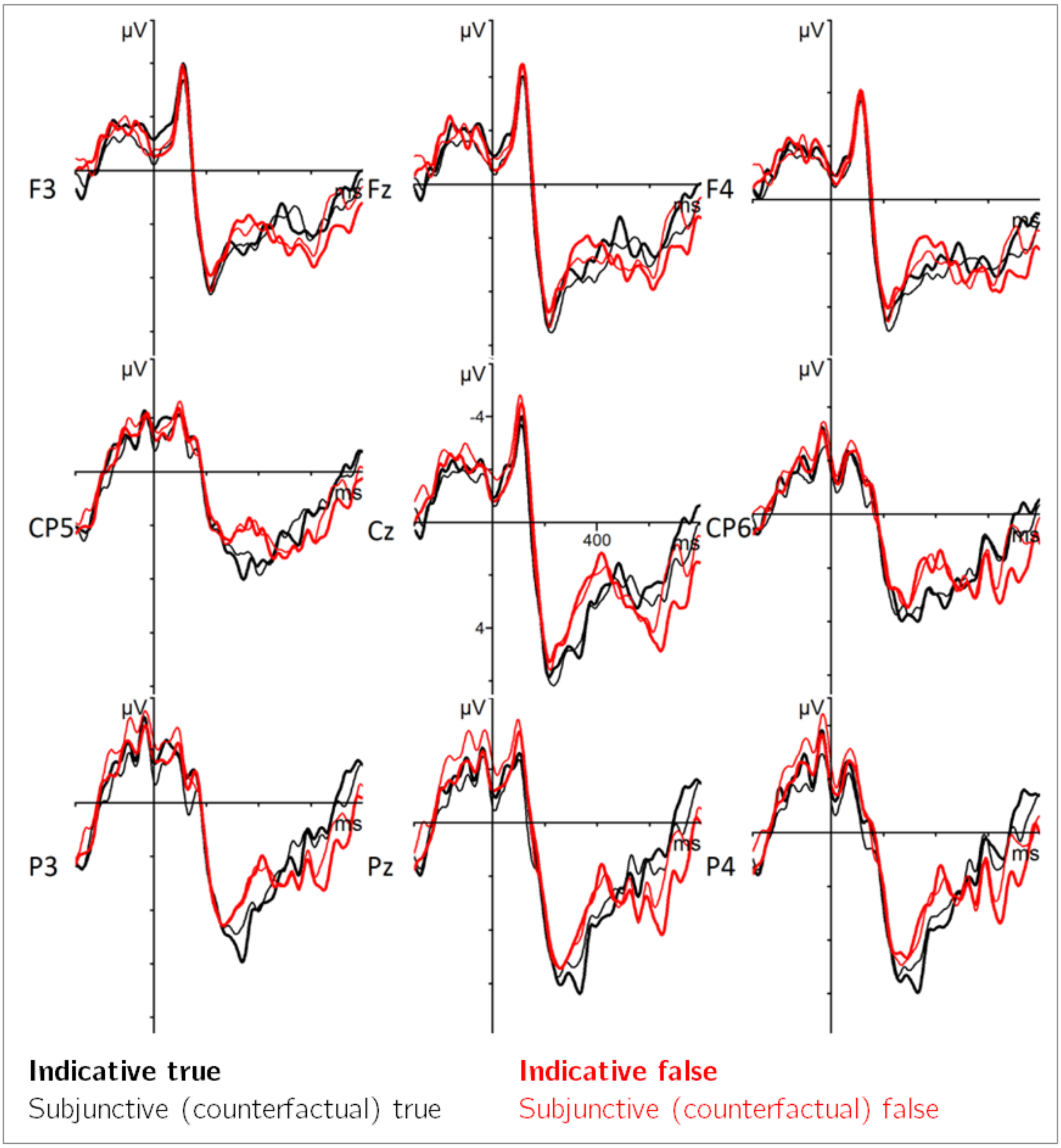

Figure 1. Grand average waveforms time-locked to the sentence-final content word establishing the manipulation of truth: false (red) and true (black) endings of indicative (thik line) and subjunctive (thin line) conditionals.

2. *Pre-critical baseline interval*

	Time window																							
	-200 - -150 ms				-150 - -100 ms				-100 - -50 ms				-50 - 0 ms				0 - 50 ms				50 - 100 ms			
	df	*F*	*p*	*sig*	*df*	*F*	*p*	*sig*	*df*	*F*	*p*	*sig*	*df*	*F*	*p*	*sig*	*df*	*F*	*p*	*sig*	*df*	*F*	*p*	*sig*
ANOVA lateral electrodes																								
MOOD	1,15	1.78	.20		1,15	< 1	.42		1,15	< 1	.54		1,15	< 1	.47		1,15	< 1	.51		1,15	1.15	.30	
AUX	1,15	< 1	.52		1,15	< 1	.99		1,15	1.01	.33		1,15	< 1	.48		1,15	< 1	.58		1,15	3.04	.10	
ROI	5,75	5.66	< .01	**	5,75	5.26	<.01	**	5,75	7.73	< .001	***	5,75	10.45	< .001	***	5,75	1.40	.26		5,75	2.30	.13	
MOOD*AUX	1,15	< 1	.54		1,15	< 1	.74		1,15	2.26	.15		1,15	< 1	.41		1,15	2.00	.18		1,15	3.93	.07	
MOOD*ROI	5,75	2.39	.09		5,75	1.39	.26		5,75	1.87	.14		5,75	1.97	.12		5,75	< 1	.53		5,75	< 1	.48	
AUX*ROI	5,75	3.04	< .05	*	5,75	< 1	.46		5,75	1.50	.24		5,75	1.39	.26		5,75	1.20	.32		5,75	1.72	.20	
MOOD*AUX*ROI	5,75	< 1	.42		5,75	< 1	.50		5,75	< 1	.53		5,75	< 1	.96		5,75	< 1	.52		5,75	< 1	.47	
ANOVA midline electrodes																								
MOOD	1,15	2.79	.12		1,15	< 1	.44		1,15	< 1	.74		1,15	< 1	.64		1,15	< 1	.40		1,15	1.61	.22	
AUX	1,15	< 1	.66		1,15	< 1	.91		1,15	1.18	.29		1,15	< 1	.43		1,15	< 1	.37		1,15	4.07	.06	
MID	2,30	2.99	.09		2,30	3.94	<. 05	*	2,30	3.26	0.08		2,30	11.19	< .01	**	2,30	< 1	.59		2,30	< 1	.96	
MOOD*AUX	1,15	1.76	.20		1,15	< 1	.41		1,15	1.23	.29		1,15	< 1	.50		1,15	1.63	.22		1,15	5.85	< .05	*
MOOD*MID	2,30	< 1	.36		2,30	< 1	.36		2,30	< 1	.46		2,30	< 1	.72		2,30	2.62	.09		2,30	< 1	.43	
AUX*MID	2,30	3.56	.07		2,30	< 1	.95		2,30	< 1	.55		2,30	1.50	.24		2,30	< 1	.38		2,30	< 1	.38	
MOOD*AUX*MID	2,30	< 1	.52		2,30	< 1	.56		2,30	< 1	.46		2,30	< 1	.40		2,30	< 1	.85		2,30	< 1	.66	

Table 2. Test-statistics of 50 ms baseline intervals around the critical auxiliary disambiguating mood. AUX = auxiliary, AL = anterior left, AR = anterior right, CL = central left, CR = central right, PL = posterior left, PR = posterior right.

Resolving the AUX*ROI interaction in the -200 to -100ms time window did not reveal any ROI in which the effect of AUX was significant (AL/CL/CR: T(15)<1, AR: T(15) = 1.16, p_{FDR} = .36, PL: T(15) = 1.04, p_{FDR} = .63, PR: T(15) = 1.89, p_{FDR} = .36)

Resolving the MOOD*AUX interaction in the 50-100ms time window revealed that the difference between verbs (to have vs. to be) was only significant in indicative (T(15)=3.49, p_{FDR} <.01) but not subjunctive mood (T(15)<1).

3. *Original German stimulus sentences (vignettes) with English translation (analogous):*

Wenn die Abgase gefiltert waren/wären, dann war/wäre die Luft ungiftig/vergiftet.
If the exhaust gases were/had been filtered, then the air was/would have been nontoxic/toxic.
Wenn die Antworten gestimmt hatten/hätten, dann waren/wären die Noten gut/schlecht.
If the answers were/had been right, then the grades were/would have been good/bad.
Wenn die Äpfel gereift waren/wären, dann waren/wären sie süß/sauer.
If the apples were/had been ripe, then they were/would have been sweet/sour.
Wenn die Bananen unreif waren/wären, dann waren/wären sie grün/braun.
If the bananas were/would have been unripe, then they were/would have been green/brown.
Wenn die Blumen geblüht hatten/hätten, dann waren/wären die Felder bunt/kahl.
If the flowers were/had been blooming, then the fields were/would have been colorful/naked.
Wenn die Blumen geduftet hatten/hätten, dann waren/wären sie echt/unecht.
If the flowers were/had been fragrant, then they were/would have been real/artificial.
Wenn die Bremsen gequietscht hatten/hätten, dann waren sie ungeschmiert/geschmiert.
If the brakes were/had been squeaking, then they were/would have been unoiled/oiled.
Wenn die Briefe geöffnet waren/wären, dann waren/wären sie aufgerissen/versiegelt.
If the letters were/would have been opened, then they were/would have been ripped/sealed.
Wenn die Chips aufgegessen waren/wären, dann waren/wären die Tüten leer/voll.
If the crisps were/had been eaten up, then the bags were/would have been empty/full.
Wenn die Christen gebeichtet hatten/hätten, dann waren/wären sie geläutert/verdammt.
If the Christians (had) confessed, then they were/would have been refined/condemned.
Wenn die Demokraten gewonnen hatten/hätten, dann waren/wären die Republikaner traurig/glücklich.
If the Democrats (had) won, then the Republicans were/would have been sad/happy.
Wenn die Eltern besorgt waren/wären, dann waren sie unruhig/ruhig.
If the parents were/had been worried, then they were/would have been agitated/calm.
Wenn die Feinde besiegt waren/wären, dann waren/wären die Soldaten erfolgreich/erfolglos.
If the enemies were/had been defeated, then the soldiers were/would have been successful/unsuccessful.
Wenn die Felder bestellt waren/wären, dann waren/wären die Bauern zufrieden/unzufrieden.
If the crops were/had been harvested, then the peasants were/would have been content/discontent.
Wenn die Fenster geöffnet waren/wären, dann war/wäre die Zimmerluft frisch/stickig.
If the windows were/had been opened, then the air was/would have been fresh/sticky.
Wenn die Fische gefüttert waren/wären, dann waren/wären sie satt/hungrig.
If the fish were/had been fed, then they were/would have been full/hungry.
Wenn die Freunde gratuliert hatten/hätten, dann waren/wären sie höflich/unhöflich.
If the friends (had) congratulated, then they were/would had been polite/rude.
Wenn die Gäste getanzt hatten/hätten, dann war/wäre die Tanzfläche gefüllt/leer.
If the guests were/had been dancing, then the dance floor was/would have been full/empty.
Wenn die Getränke gezuckert waren/wären, dann waren/wären sie süß/bitter.
If the drinks were/had been sugared, then they were/would have been sweet/bitter.
Wenn die Häuser gestrichen waren/wären, dann waren sie hübsch/schäbig.
If the houses were/had been painted, then they were/would have been pretty/shabby.
Wenn die Hunde gejault hatten/hätten, dann waren/wären sie laut/leise.
If the dogs (had) whined, then they were/would have been loud/quiet.
Wenn die Katzen gegessen hatten/hätten, dann waren/wären sie satt/hungrig.
If the cats (had) eaten, then they were/would have been full/hungry.
Wenn die Kerzen gebrannt hatten/hätten, dann war/wäre das Wachs weich/hart.
If the candles (had) burned, then the wax was/would have been soft/hard.
Wenn die Kinder erzogen waren/wären, dann waren/wären die Großeltern zufrieden/unzufrieden.
If the children were/had been well-behaved, then the grandparents were content/discontent.
Wenn die Kinder gespielt hatten/hätten, dann waren/wären ihre Finger schmutzig/sauber.
If the children were/had been playing, then their hands were/would have been dirty/clean.
Wenn die Kollegen verreist waren/wären, dann waren/wären die Büros leer/voll.
If the colleagues were/had been on holidays, then the office was/would have been empty/crowded.
Wenn die Lampen gebrannt hatten/hätten, dann waren/wären sie warm/kalt.
If the lamps were/had been switched on, then they were/would have been warm/cold.
Wenn die Männer gesoffen hatten/hätten, dann waren/wären sie betrunken/nüchtern.
If the men were/had been drinking heavily, then they were/would have been drunk/sober.

Wenn die Mörder gestanden hatten/hätten, dann waren/wären sie einsichtig/uneinsichtig.
If the murderers (had) confessed, then they (would have) admitted their guilt/remained unwilling to see their error.
Wenn die Nägel geschnitten waren/wären, dann waren sie kurz/lang.
If the nails were/had been trimmed, then they were/would have been short/long.
Wenn die Patienten geheilt waren/wären, dann waren/wären sie gesund/krank.
If the patients were/had been healed, then they were/would have been healthy/sick.
Wenn die Pfeile getroffen hatten/hätten, dann waren/wären die Schützen gut/schlecht.
If the arrows (had) hit, then the archers were/would have been good/bad.
Wenn die Pilger gefastet hatten/hätten, dann waren/wären sie hungrig/satt.
If the pilgrims (had) fasted, then they were/would have been hungry/full.
Wenn die Piloten gestreikt hatten/hätten, dann war/wäre der Flugverkehr beeinträchtigt/unbeeinträchtigt.
If the pilots were/had been on strike, then the air traffic was/would have been impaired/unimpaired.
Wenn die Preise gestiegen waren/wären, dann waren/wären sie höher/tiefer.
If the prices rose/had risen, then they were/would have been higher/lower.
Wenn die Räder aufgepumpt waren/wären, dann waren/wären sie hart/weich.
If the wheels were/had been pumped up, then they were/would have been hard/soft.
Wenn die Raketen getroffen hatten/hätten, dann waren/wären die Häuser zerstört/unbeschädigt.
If the missiles (had) hit, then the houses were/would have been damaged/unharmed.
Wenn die Schaufenster zerschlagen waren/wären, dann war/wäre das Glas zerbrochen/unbeschädigt.
If the display windows were/had been smashed, then the pane was/would have been broken/unharmed.
Wenn die Scheiben beschlagen waren/wären, dann war/wäre die Sicht schlecht/klar.
If the windows were/had been fogged, then the view was/would have been bad/clear.
Wenn die Schnitzel gebraten waren/wären, dann waren/wären sie gar/roh.
If the schnitzels were/had been roasted, then they were/would have been done/raw.
Wenn die Schüler gelernt hatten/hätten, dann waren/wären sie klüger/dümmer.
If the pupils (had) studied, then they were/would have been smarter/more stupid.
Wenn die Schüler gequatscht hatten/hätten, dann waren/wären sie unaufmerksam/aufmerksam.
If the pupils were/had been chatting, then they were/would have been inattentive/attentive.
Wenn die Schwestern telefoniert hatten/hätten, dann waren/wären sie informiert/uninformiert.
If the sisters (had) talked on the phone, then they were/would have been updated/uninformed.
Wenn die Söhne geraucht hatten/hätten, dann waren/wären die Eltern wütend/glücklich.
If the sons (had) smoked, then the parents were/would have been angry/happy.
Wenn die Speisen geschmeckt hatten/hätten, dann waren/wären die Gäste zufrieden/unzufrieden.
If the meals (had) tasted good, then the guests were/would have been pleased/discontent.
Wenn die Straßen beleuchtet waren/wären, dann waren/wären die Wege hell/dunkel.
If the streets were/had been well-lit, then the paths were illuminated/dark.
Wenn die Tabletten gewirkt hatten/hätten, dann waren/wären die Schmerzen schwächer/stärker.
If the pill took/had taken effect, then the pains were/would have been weaker/stronger.
Wenn die Urkunden unterzeichnet waren/wären, dann waren/wären sie gültig/ungültig.
If the certificates were/had been signed, then they were/would have been valid/invalid.
Wenn die Waren ausverkauft waren/wären, dann waren/wären die Regale leer/voll.
If the goods were/had been sold out, then the shelves were/had been empty/full.
Wenn die Wolken verzogen waren/wären, dann war/wäre der Himmel blau/grau.
If the clouds were/had been gone, then the sky was/would have been blue/gray.
Wenn die Würfel gezinkt waren/wären, dann war/wär das Spiel unfair/fair.
If the dice were/had been rigged, then the game was/would have been unfair/fair.
Wenn die Zuhörer geklatscht hatten/hätten, dann waren/wären die Vortragenden zufrieden/unzufrieden.
If the audience (had) applauded, then the speakers were/would have been content/discontent.
